# Improved asthma control in patients with severe, persistent allergic asthma after 12 months of nightly temperature-controlled laminar airflow: an observational study with retrospective comparisons

**DOI:** 10.3402/ecrj.v2.28531

**Published:** 2015-07-29

**Authors:** Uwe Schauer, Karl-Christian Bergmann, Michael Gerstlauer, Sylvia Lehmann, Monika Gappa, Amelie Brenneken, Christian Schulz, Peter Ahrens, Jens Schreiber, Michael Wittmann, Eckard Hamelmann, all members of the German Asthma Net (GAN)

**Affiliations:** 1 Allergy-Center-Ruhr, University Children's Hospital, Ruhr University, Bochum, Germany; 2 Allergy-Center Charité, Charité-Universitätsmedizin, Berlin, Germany; 3 Department of Pediatric Pneumology and Allergology, Children's Hospital Augsburg, Augsburg, Germany; 4 Department of Pediatric Pneumology, Allergology and Immunology, University Hospital Aachen, Aachen, Germany; 5 Center for Children and Adolescent Care, Marien Hospital, Wesel, Germany; 6 Department of Pneumology, University Hospital Regensburg, Regensburg, Germany; 7 Department of Pulmonology and Allergology, Pediatric Clinics, Darmstadt, Germany; 8 Department of Pneumology, University Hospital Magdeburg, Magdeburg, Germany; 9 Department of Pneumology, Hospital Bad Reichenhall, Bad Reichenhall, Germany; 10 Children's Center Bethel, Evangelisches Krankenhaus Bielefeld, Bielefeld, Germany

**Keywords:** antiasthmatic therapy, allergic rhinitis, exacerbations, airflow therapy, resource use, allergen avoidance, environmental control

## Abstract

**Introduction:**

Continuous or episodic allergen exposure is a major risk factor of frequent symptoms and exacerbations for patients with allergic asthma. It has been shown that temperature-controlled laminar airflow (TLA) significantly reduced allergen exposure and airway inflammation and improved quality of life of patients with poorly controlled allergic asthma.

**Objective:**

The objective was to evaluate the effects of nighttime TLA when used during real-life conditions for 12 consecutive months in addition to the patients’ regular medication.

**Methods:**

This multicenter, pre- and postretrospective observational study included patients with inadequately controlled moderate-to-severe allergic asthma who received add-on treatment with TLA for 12 consecutive months. Data on medication use, asthma control, asthma symptoms, lung function, use of hospital resources, and exacerbations were collected after 4 and 12 months and compared with corresponding data collected retrospectively from medical records during the year prior to inclusion in the study.

**Results:**

Data from 30 patients (mean age 28; range 8–70) completing 4 months and 27 patients completing 12 months of TLA use are presented. The mean number of exacerbations was reduced from 3.6 to 1.3 (*p*<0.0001), and the ratio of asthma-related emergency room visits or hospitalizations diminished from 72.4 to 23.3% (*p*=0.001) or from 44.8 to 20.0% (*p*<0.05), respectively, after 12 months of TLA use. The Asthma Control Test index increased from 14.1 to 18.5 (*p*<0.0001). After 4 months of TLA use, clear improvements can be shown for most variables in line with the data collected after 12 months.

**Conclusions:**

The addition of TLA to the patients’ regular medication significantly reduced exacerbations, asthma symptoms, and the utilization of hospital resources. The data support that TLA may be an important new non-pharmacological approach in the management of poorly controlled allergic asthma.

Asthma is a chronic inflammatory disease associated with genetic predisposition and increased responsiveness to multiple triggers inducing and maintaining airway inflammation (1). In allergic asthma, this airway inflammation is mainly driven by the exposure to inhalant allergens to which the patient is sensitized.

Accordingly, reduction of allergen exposure is recommended to reduce asthma symptoms in addition to appropriate antiinflammatory medications (2–6). Patients with persistent allergic asthma are recommended to minimize the exposure to inhalant allergens to improve asthma control (7, 8).

With this approach, asthma control can be attained and maintained for the majority of patients in a controlled trial setting, but this may be different in real-life practice (9–14). Poorly controlled asthma accounts for a disproportionately high share of the costs of asthma and represents a heavy socioeconomic burden (15–18). A subgroup of these patients with severe and persistent asthma even needs oral corticosteroids (OCS) as maintenance or intermittent treatment despite the well-documented long-term side effects (19).

The patient group with unstable severe allergic asthma has a high unmet medical need and is at increased risk of hospitalization for exacerbations and for asthma death underlining the requirement for further means to gain control (20).

Air filter devices have been tested to achieve a reduction in allergen exposure. Blinded studies have, however, failed to demonstrate significant benefit, which indicates that the reduction in allergen exposure achieved by these techniques was insufficient to impact airway inflammation and asthma symptoms (21, 22).

The device Airsonett™ (Airsonett AB, Ängelholm, Sweden) uses a temperature-controlled laminar airflow (TLA) of purified air directed to the breathing zone of a patient during sleep. The device, below referred to as TLA, distributes a filtered cooled laminar airflow, descending from an overhead position, which displaces aeroallergens from the breathing zone (23).

The efficacy of the TLA device has previously been demonstrated in a randomized, controlled, parallel-group trial that evaluated the add-on use of the device or a placebo device in 312 patients with persistent atopic asthma and features of inadequate asthma control, according to Global Initiative for Asthma (GINA) 2006 (24). Nocturnal control of aeroallergen exposure was shown to improve quality of life and to reduce airway inflammation in adults and children without significant adverse effects (25). The most pronounced beneficial effects were seen in those with highest asthma treatment intensity at baseline (GINA 4) and in those with poor asthma control at baseline.

The aim of this study was to investigate if the use of the TLA device during nighttime, as an add-on to prevailing regular asthma medication, would reduce airway inflammation and improve symptoms, including exacerbations, hospital admissions, emergency visits, and the use of systemic corticosteroids in patients with poorly controlled moderate-to-severe persistent allergic asthma. Of special interest was the evaluation of the frequency of asthma exacerbations and other consequences, for example, OCS use, hospital admissions or emergency room (ER) visits, due to improved disease management under the influence of TLA application.

## Methods

### Ethics

The study was approved by the Ethics Committee (der Med. Fakultät Bochum) on November 4, 2011 (Ref No. 4175–11). Local management (R&D) approval was obtained at each center.

### Patients

Potential eligible patients were identified at participating sites based on the below mentioned screening and eligibility criteria from a clinic database or diary. Informed patient consent was sought for study participation as well as research access to the medical records and, if needed, researcher contact with the general practitioner to complete the study dataset. For underage patients, written informed consent was also collected from their guardians.

### Study design

A non-randomized uncontrolled pre–post retrospective observational study was carried out to investigate the effect of 12 months’ TLA use during real-life conditions. The study was performed in 10 German centers specialized in pediatric or adult lung diseases with a special interest in severe and difficult-to-treat asthma. Patients with severe and difficult-to-control persistent asthma were followed according to a structured study protocol with clinical visits after 4 and 12 months while on nightly TLA use. The TLA device was installed in the home of the patients within 10 days of study inclusion.

Prospectively collected data covering the 12 months of TLA use on asthma exacerbations and emergency care visits were compared with corresponding data collected retrospectively for the study baseline from medical records at the hospitals or clinics where the patients had been treated during the prestudy year.

There was no prespecified hypothesis that the study was statistically powered to investigate. As the study involved a broad group of patients with poorly controlled persistent asthma, and the management of the patients during the study period was deemed by the individual status of the patient triggered by the same treatment goals during regular medical care, the handling of patients and clinical decisions were not restricted in any way, as normal in controlled clinical trials. The study, therefore, intentionally compared treatment effects in real-life circumstances.

### Study participants

Specific screening criteria for potential study patients were applied at all participating centers (Row 1 in Table 1). Study participants were recruited during the period December 2011 to November 2012.

**Table 1:** Inclusion criteria for screening and recruitment (patients were required to fulfill each of the inclusion criteria 2–5)

1:	Screening criteria for potential study participation	Criteria for severe asthma, based on the German Asthma Net (27) At least one severe exacerbation during the past 12 months which fulfilled at least one of the following criteria: Inpatient treatment or treatment in an emergency department Exacerbation treated outside the hospital with systemic steroids for at least 3 days or by an increasing dose of a systemic therapy for at least 3 days Willingness to participate in the study where baseline data will be collected at study inclusion, that is, before the TLA use, after 4 months (TLA treatment success evaluation) and after 12 months use (long-term evaluation).
2:	Inclusion criteria for severe asthma (26)	The study population consisted of three subgroups of patients, those with: Untreated, severe asthma Difficult-to-treat severe asthma Treatment-resistant severe asthma, including: Asthma for which control is not achieved despite the highest level of recommended treatment: refractory asthma and corticosteroid-resistant asthma; or Asthma for which control can be maintained only with the highest level of recommended treatment.
3:	Inclusion criteria for positive bronchial reversibility and positive bronchial hyperreactivity (BHR)	Positive bronchial reversibility test (≥12% increase in FEV1 after short-acting beta2 agonists (SABA); or Significant bronchial hyperreactivity (BHR) following unspecific provocation (e.g. with methacholine or treadmill). A positive BHR was defined according to the following (31): A decrease in FEV1>20% after methacholine challenge test; or A decrease in FEV1>10% after a standardized exercise bronchial provocation test; or Circadian PEF variability>20% over a period of 3–14 days, derived from at least four measurements per day.
4:	Inclusion criteria for high level of therapy	Long-term therapy with high-dose ICS (only for children and adolescents aged 6–18) (>400 µg budesonide equivalent/>200 µg fluticasone alone); or Daily therapy with medium-to-high-dose ICS (≥400 µg budesonide equivalent/≥200 µg fluticasone) in combination with long-acting beta2 agonists and/or leukotriene receptor antagonist and/or theophylline; or Therapy with OCS fixed ≥3 past months.
5:	Inclusion criteria for inadequate asthma symptom control according to NVL (27) in the past 4 weeks prior to inclusion	Asthma symptoms ≥3 times per week; or Use of rescue medication; or Limited activity due to asthma; or Any symptoms at night; or Exacerbation(s) ≥1 last year, treatment with systemic steroids and/or hospitalization required; or Reduced lung function: pathological FEV1/FVC ratio or FEV1 at inclusion.
6:	Outcome parameter: The ATS/ERS definition of moderate and severe asthma exacerbations (30)	A moderate asthma exacerbation should result in a temporary change in treatment, in an effort to prevent the exacerbation from becoming severe, and should include one or more of the following: Deterioration in symptoms, deterioration in lung function, and increased rescue bronchodilator use. These features should last for 2 days or more but not be severe enough to warrant systemic corticosteroid use and/or hospitalization. ER visits for asthma (e.g. for routine sick care), not requiring systemic corticosteroids, may be classified as moderate exacerbations. Use of oral corticosteroids, or an increase from a stable maintenance dose, for at least 3 days. The magnitude of change in these outcomes differs depending on the population studied and each individual patient's baseline variation.

Patients with severe difficult-to-control asthma (Row 2 in Table) fulfilling the following *eligibility criteria* were recruited (26): Physician diagnosis of asthma, difficult-to-treat asthma, good treatment adherence and trained inhalation technique, positive bronchodilator response and/or bronchial hyperreactivity (BHR; Row 3 in Table 1), high level of control medication (Row 4 in Table 1), inadequate asthma symptom control according to National guidelines (27), during the past 4 weeks (Row 5 in Table 1), written informed consent signed by patient and/or guardians, hospital medical records available and accessible.

The following *ineligibility criteria* were applied: Diagnosis of other obstructive or systemic lung diseases (e.g. cystic fibrosis, COPD) at the time of inclusion, other lung diseases or congenital malformations in the airways, other significant chronic conditions, congenital or acquired heart disease with significant functional changes.

### Data collection

Retrospective clinical data according to the study case report form (CRF), for the 12-month period preceding study inclusion and start of the period with the TLA device, were collected from paper or electronic hospital medical records available at the clinics where the patients had visited or been treated in the previous year.

Data collected at study inclusion (baseline data) included patient demographics (age, sex, and BMI), presence of concomitant allergic diseases (rhinitis, eczema, and food allergies), concomitant allergy/asthma medications, number of perennial allergies, lung function parameters (PEF, FEV1, and FEV1%), classification of asthma control (27); Table 2), the Asthma Control Test (28); ACT index), symptoms of BHR, daily or nightly asthma symptoms, and inability to work (or go to school) due to the asthma.

**Table 2:** Criteria for evaluation of asthma control based on The Global Strategy for Asthma Management and Prevention, Global Initiative for Asthma (GINA) (24); information related to any week within the past 4 weeks

Criterion	Controlled asthma (all criteria fulfilled)	Partly controlled asthma (1–2 criteria within 1 week fulfilled)	Uncontrolled asthma
Symptoms during the day	None or ≤2 times per week	>2 times per week	At least three criteria from ‘Partly controlled Asthma’ – within 1 week
Restriction of activities in everyday life	None	Yes
Nocturnal symptoms/at awakening	None	Yes
Intake of a rescue medication/emergency treatment	None or ≤2 times per week	>2 times per week
Lung function (PEF or FEV1)	Normal	<80% of the predicted or personal best value	
Exacerbation^a^	None	One or more per year	One per week

a Any exacerbation in a week is defined as uncontrolled asthma. Definition of exacerbation: Episode with increase in shortness of breath, coughing, wheezing, and/or chest tightness, that goes along with a decrease of PEF or FEV1.

The Childhood Asthma Control Test™ (29) was used for patients under 14 years of age.

At baseline, retrospective information from hospital medical records related to exacerbations during the 12 months prestudy period was collected including the number of exacerbations and need for medical intervention, for example, asthma-related ER visits, asthma-related hospital admissions, and exacerbations requiring intensive care.

For classification of exacerbations, the ATS/ERS definition of moderate and severe exacerbations was used (Row 6 in Table 1; 30).

After 4 months (16 weeks) and 12 months of TLA use, the data corresponding to that at study baseline were recorded. Data about exacerbations and need for hospital/medical resources during the past 4 or 8 months were collected at the study visits after 4 and 12 months, respectively.

Data were collected in a paper CRF and entered into an Excel-based study database after study completion and analyzed according to a prespecified analysis plan.

### Primary outcomes

The primary objective of the study was to evaluate the intraindividual change in asthma control after 12 months of TLA use: 1) the number of exacerbations; 2) the need of asthma-related emergency care; 3) the need of asthma-related hospital admissions; 4) the need of asthma-related intensive care; 5) the use of OCS; 6) changes in asthma control according to ACT index and GINA classification.

### Secondary outcomes

Lung function, use of relievers, ability to work (or go to school), symptoms of BHR, and frequencies of daily or nightly symptoms were documented as secondary outcome parameters.

### Statistics

Patient demographics and other baseline characteristics were summarized using descriptive statistics, contingency tables for qualitative variables; and mean, standard deviation, median, minimum, and maximum for quantitative variables. The analysis used information for all patients with collected postuse data in accordance with an Intention-to-treat approach. No imputations of missing values were applied. Data collected after 4 months of TLA use, related to exacerbations and requirements of medical resources due to exacerbations, were compared with corresponding data collected at baseline. All statistical tests were performed on the observed differences between post-TLA and pre-TLA values. The changes in exacerbations and ACT score were analyzed using the Wilcoxon Signed Rank test. All qualitative variables were analyzed using McNemar's test.

## Results

From 10 participating hospitals, patients were actively recruited at eight centers. A total of 43 patients were found to be eligible for participation and were screened. Thirty-two patients (74%) consented to study participation: 27 were classified with difficult-to-control asthma and 5 with treatment-resistant asthma. Two of these thirty-two patients never showed up for the follow-up visits after 4 and 12 months, and another three patients did not show up for the final visit after 12 months of TLA use. Data at 4 months were completed for 30 patients and at 12 months for 27 patients.

### Baseline demography and patient characteristics

Patient baseline characteristics are presented in Table 3. Patient mean age was 28, and 50% were <18 years of age at baseline. The mean number of exacerbations during the previous 12 months was 3.6 (range 0–12); only four patients (13%) had no exacerbation at all during the prestudy period. According to the criteria for evaluation of asthma control, 16 patients (55%) had uncontrolled asthma and 10 (34%) partly controlled asthma. The mean ACT score was 14.1. In addition to the diagnosis of allergic asthma, the great majority of patients also showed at least one other form of allergic symptom as allergic rhinitis, eczema, or food allergy. The ratio with present symptoms of BHR was 73%. Ten patients (33%) were on regular treatment with OCS at the beginning of the study and 13 (43%) with anti-IgE monoclonal antibodies.

**Table 3:** Patient baseline characteristics before TLA use

Patient characteristics at inclusion	Mean (SD); range	Proportion (%)
Age	28.1 (20.0); 8.3–70.9	
Female		16/30 (53)
BMI	21.8 (4.4); 13.7–30.9	
Concomitant allergic rhinitis		23/30 (76)
Concomitant allergic eczema		10/30 (33)
Number of different types^a^ of perennial allergies (dust mites/pet dander/mold/other)	2.0 (1.0); 0–4	
Specific food allergy		11/30 (37)
Number of exacerbations during the previous 12 months prior to inclusion	3.6 (3.5); 0–12	
Asthma control (GINA)		
Uncontrolled		16/29 (55)
Partly controlled		10/29 (34)
Controlled		3/29 (10)
Asthma control test (ACT) score^b^	14.1 (6.6); 5–27	
FEV1(l) (mean and range)	1.92^c^ (0.73–3.44)	
FEV1/FVC (%); (mean and range)	79.2^d^ (42–120)	
PEF (l/min) (mean and range) [l/min]	4.22 (1.26–6.13)	
Medication		
ICS [DDD:s µg/day]^e^	1,095 (833.8)	
Daily OCS treatment (mg)^e^	33.5; 10–100	10/30 (33)
Anti-IgE treatment (mg per dose)^f^	150–600	13/30 (43)

a Four subgroups/classes of perennial allergies were considered (dust mites/pet dander/mold/other). A patient thus could get up to four points if allergic to one or more allergens in each of the four groups.

b For one child, the adult version was completed at first visit, for another two children, the adult version was completed at the last visit.

c Two missing values.

d Three missing values.

e The doses used by the OCS users, 10/30 (33%).

f The doses used by the anti-IgE users, 13/30 (43%).

### Exacerbations

The screening criteria for study participation required at least one episode of severe exacerbation during the previous 12 months; four patients did not fulfill this requirement and were excluded for statistical analysis for this parameter. One patient had no previous exacerbations at all and another three patients had had previous exacerbations but not during the past 12 months.

**Fig. 1:**
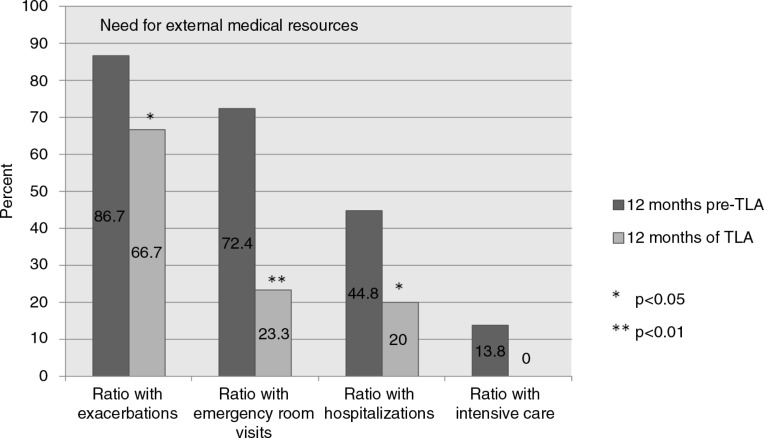
Ratio (%) of patients studied with asthma exacerbations during the previous 12 months requiring emergency room visits, inpatient hospitalizations, or intensive care at baseline and after 12 months TLA use.

During the 12 months of TLA use, the exacerbation frequency diminished from an average of 3.6 (range 1–12) to an average number of exacerbations of 1.3 for the whole period (range 0–5; *p*<0.001). The proportion of patients without any exacerbations increased from 13 to 33% (*p*<0.05) during the TLA period (Table 4 and Fig. 1).

**Table 4:** Main study outcomes at baseline and after 4 and 12 months of TLA use (statistical comparisons with baseline values)

Ratios (*n*/*N*;%) or means (m; range) and *p*	Visit 1 Baseline	Visit 2 4 months TLA	Visit 3 12 months TLA
Number of exacerbations in the past 12 months *(NB: Data at Visit 2 refers to last 4 months)*	3.6 (1–12)	0.7 (0–4) (<0.0001)	1.3 (0–5) (<0.0001)
Ratio with no exacerbations in the past 12 months *(NB: Data at Visit 2 refers to last 4 months)*	4/30; 13.3%	18/30; 60.0% (0.0002)	10/30; 33.3% (0.0143)
Ratio requiring ≥1 asthma-related emergency room visits in the past 12 months *(NB: Data at Visit 2 refers to last 4 months)*	21/29; 72.4%	4/29; 13.8% (0.0002)	7/30; 23.3% (0.0010)
Ratio requiring ≥1 asthma-related inpatient hospitalizations in the past 12 months *(NB: Data at Visit 2 refers to last 4 months)*	13/29; 44.8%	3/29; 10.3% (0.0027)	6/30; 20.0% (0.0193)
Ratio requiring intensive care in the past 12 months *(NB: Data at Visit 2 refers to last 4 months)*	4/29; 13.8%	0	0 (>0.05)
Ratio of patients on oral corticosteroids	10/30; 33.3%	7/30; 23.3% (0.0833)	6/27; 22.2% (0.1025)
Asthma Control Test index	14.1^a^ (5–27)	17.8 (8–25) (0.0023)	18.5 (8–27) (<0.0001)
Ratio with present symptoms of BHR	22/30; 73.3%	14/29; 48.3% (0.0455)	8/27; 33.3% (0.0045)
Ratio with uncontrolled asthma; GINA	16/29; 55.2%	6/30; 20.0% (0.00503)	0 (0.0003)
Ratio with controlled asthma; GINA	3/29; 10.3%	8/30; 26.7%	9/27; 33.3%
Ratio with incapacity to work/attend school	13/30; 43.3%	8/29; 27.6% (0.0956)	6/27; 22.2% (0.1573)
FEV1	1.92^b^ (0.73–3.44)	2.15^b^ (0.54–4.67) (0.1661)	2.28^b^ (0.7–4.81) (0.0019)
FEV1/FVC (%)	79.2^c^ (42–120)	80.8^b^ (35–119) (0.3493)	85.0^b^ (49–117) (0.1091)
PEF	4.22^c^ (1.26–6.13)	4.96^d^ (1.25–10.0) (0.6373)	5.47^c^ (1.2–10.48) (0.0546)

a One missing value.

b Two missing values.

c Three missing values.

d Seven missing values.

Within the first 4 months of TLA use, 60% of the participants were free of exacerbations (*p*<0.001). The mean number of exacerbations during that period was 0.7 (*p*<0.0001).

### Resource use

During the 12 months of TLA use, the patient proportion needing asthma-related ER visits was reduced from 72 to 23% (*p*=0.001). The proportion of patients requiring asthma-related inpatient hospitalization declined from 45 to 20% (*p*<0.05). No patient needed intensive care treatment after TLA was introduced as compared with 14% during the previous year, but this difference was statistically not significant (Table 4 and Fig. 1).

Data collected after the first 4-month treatment period verified a fast onset of the TLA effect, with only 14% of the participants requiring an ER visit (*p*<0.001) and 10% requiring inpatient hospitalization during that period (*p*<0.01).

### Asthma control

**Fig. 2:**
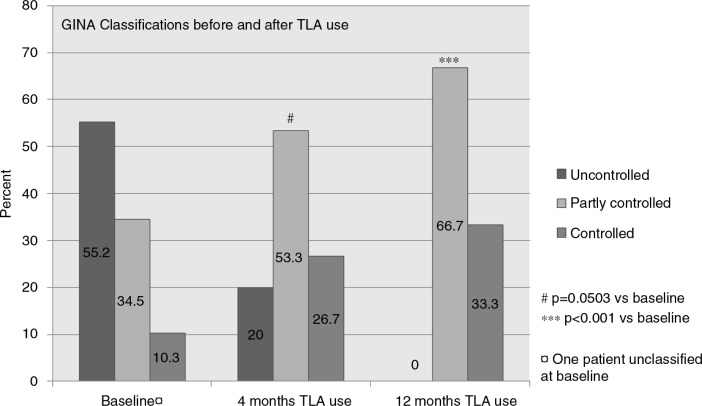
Classification of the asthma control of patients studied according to GINA (24) at baseline and after 4 and 12 months of TLA use.

After 12 months of TLA use, the proportion of patients with uncontrolled disease had diminished from 55 to 0%, and the ratio with controlled disease increased from 10 to 34%. The outcome after 4 months approached statistical significance (*p*=0.0503) but was highly significant (*p*<0.001) after 12 months TLA use (Fig. 2).

The mean ACT score increased significantly during TLA use, at 4 months from 14.1 to 17.8 (*p*<0.01) and after 12 months a score of 18.5 was recorded (*p*<0.0001; Table 4).

**Fig. 3:**
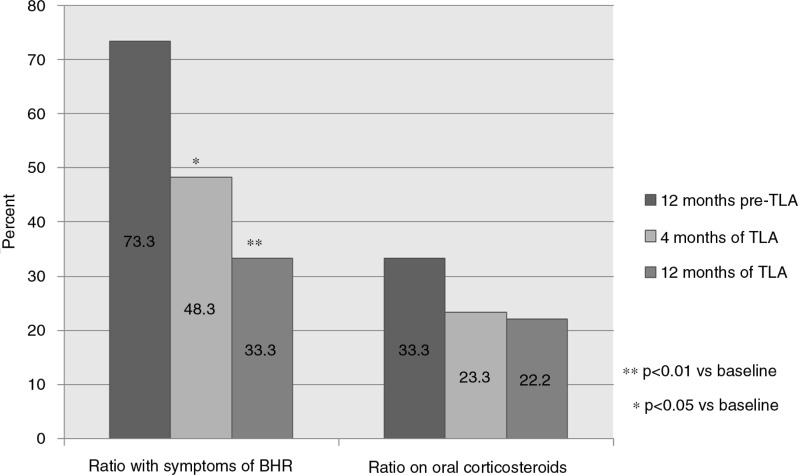
Ratio (%) of patients studied presenting symptoms of bronchial hyperreactivity (BHR) or on treatment with oral corticosteroids (OCS) at baseline and after 4 and 12 months of TLA use.

The proportion of patients with symptoms of BHR declined significantly during the study, from 73% at baseline to 33% after 12 months (*p*<0.01; Table 4 and Fig. 3).

There was a trend for daytime symptoms to decline, but this was not significant (*p*=0.09 after 12 months). However, the frequency of nighttime symptoms was significantly (*p*<0.05) lower after 4 months of TLA use; the difference approached statistical significance after 12 months (*p*=0.074).

The proportion of patients reporting an asthma-related inability to work (or go to school) was lower but did not reach significance (43–22%, Table 4).

### Concomitant medications

The proportion of patients treated with oral steroids decreased during the study from 33 to 22% (NS). After 12 months, the number of patients requiring oral CS was reduced from 10 to 6 individuals (Table 4 and Fig. 3).

There were no significant changes in the use of inhaled corticosteroids (ICS) or other regular controller medications including the omalizumab dosing.

**Fig. 4:**
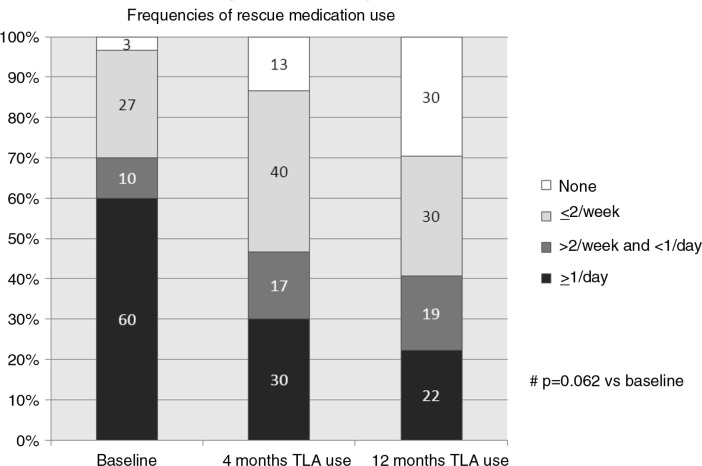
Ratios (% of all patients) with different frequencies of rescue inhalation use at baseline and after 4 and 12 months of TLA use.

The need for rescue medication, regular short-acting bronchodilators (SABA), diminished during the 12-month TLA period and approached statistical significance (*p*=0.062) (Fig. 4).

### Lung function tests

FEV1 values after 12 months of TLA improved significantly (*p*<0.01). Other values [PEF, FEV1/FVC (%)] showed numerical changes toward a normalization of lung function (Table 4).

## Discussion

To fully understand the potential impact of a novel drug or device on the quality of life of a patient, the effect in a practical, real-life setting has to be evaluated. Therefore, not only controlled clinical trials but also prospective, observational data are important (30, 32).

The long-term goals of modern asthma management should incorporate the dual components of current clinical control (e.g. symptoms, reliever use, and lung function) and future risk (e.g. exacerbations and medication side effects) (26, 30). This prospective 12-month open study demonstrates that TLA, as an add-on device to pharmacological treatment in patients with moderate-to-severe allergic asthma, significantly improved measures of current clinical control as well as reduced the risk of exacerbation and related healthcare utilization (e.g. ER visits, hospitalizations, and intensive care). Importantly, the number of patients with uncontrolled asthma was reduced from 55% at baseline to 0% after 1 year, and scores from the ACT were significantly improved with a clinically meaningful difference compared with baseline (33). The effects were apparent already after 4 months and furthermore improved and sustained over the 1-year observational study period.

These findings demonstrate that the addition of TLA add-on to standard therapy may be an effective enhancement of the management of difficult-to-control allergic asthma, supporting previous results from randomized placebo-controlled efficacy trials (25).

The study has several limitations. It was designed as a pre- and post-comparison of TLA use on the frequency of asthma exacerbations in patients with a verified medical history and documented frequent disease exacerbations requiring hospital resources, with patients serving as their own controls.

The data quality of retrospective studies relies very much on the accuracy and completeness of the clinical records referred to. Given the nature of the study, incomplete data may have been obtained from the pre-TLA period, thus rather leading to an underestimation of the effects compared with the TLA period, when the study patients and physicians were aware of the study focus on exacerbations and asthma disease deteriorations. Therefore, this study's reported reduction of the exacerbation rates and hospital resource utilizations after the TLA introduction is likely to underestimate the true differences.

Without a control group, any spontaneous improvements in asthma morbidity during the 12-month study period (TLA use) may have been missed. In addition, although we took care to select only patients with good treatment adherence, improved adherence to previous treatment during the observation period explaining part of the improvement cannot be excluded. However, we believe that this is unlikely since eligible patients had had troublesome asthma with considerable disease burden for several years, making previous non-adherence to treatment unlikely to be of major relevance.

There were some exacerbation-related parameters that did not reach formal statistical significance, nevertheless these parameters all pointed into the same direction, that is, TLA improved the asthma control status in these severely ill asthmatics. The lack of statistical significance may be attributed to the heterogeneity of the patients included and the low number of events observed.

The mode of action of TLA is to reduce the allergen exposure during sleep, which is the most likely mechanism behind the effects observed in this study. However, it should be noted that TLA also reduces the exposure to particles in the inhaled air, thus contributing to a general reduction of airway irritants (22). This may be of significant value for severely ill patients who can be expected to have considerable airway inflammation. Unfortunately, measures of airway inflammation such as exhaled nitric oxide were not part of the study protocol. As a surrogate marker for the reduction of airway inflammation after TLA introduction, we observed a significant reduction of BHR compared with baseline levels.

The effect of TLA on concomitant allergic rhinitis and/or eczema was not recorded in the study. As there are some remarkable anecdotal reports (outside this study) about effects of TLA use in asthmatic patients with eczema and rhinitis comorbidities, further studies are warranted and are now underway to investigate the effect of TLA on both these conditions.

The overall management of patients during the TLA intervention period did not change compared with the pre-TLA period. The actual nursing care during the TLA period was not restricted in any way, as is normally the case in controlled clinical trials. Consequently, the present study can be viewed as reflecting real-life circumstances and assessing symptom-related meaningful data.

## Conclusions

In conclusion, this open study assessing the effect of 12 months of TLA as add-on to pharmacological management in patients with uncontrolled moderate-to-severe allergic asthma showed a significant reduction of exacerbations and use of resources. Improvements in both subjective and objective asthma control measures confirmed the potential value of TLA in the management of patients with difficult-to-treat allergic asthma. This is even more relevant, as these severely affected patients were already treated with high doses of available medications including systemic corticosteroids. The use of TLA is not associated with any pharmacological side effects and is safe to use. Furthermore, the reduction in asthma-related hospital admissions and emergency resource use has important socioeconomic advantages. Further studies are warranted to confirm whether reductions in daily OCS use can be achieved and sustained. Finally, careful investigations should be carried out to identify those patients who will benefit most from TLA as a novel tool in the management of difficult allergic asthma.
